# A mononuclear nine-coordinated Dy(iii) complex exhibiting field-induced single-ion magnetism behaviour†

**DOI:** 10.1039/d2ra02260e

**Published:** 2022-05-10

**Authors:** Biao Hu, Jing Xi, Peipei Cen, Yan Guo, Yi Ding, Yuanyuan Qin, Yi-Quan Zhang, Xiangyu Liu

**Affiliations:** State Key Laboratory of High-efficiency Utilization of Coal and Green Chemical Engineering, College of Chemistry and Chemical Engineering, Ningxia University Yinchuan 750021 China xiangyuliu432@126.com yiding@nxu.edu.cn; College of Public Health and Management, Ningxia Medical University Yinchuan 750021 China; Jiangsu Key Laboratory for NSLSCS, School of Physical Science and Technology, Nanjing Normal University Nanjing 210023 China zhangyiquan@njnu.edu.cn

## Abstract

A new mononuclear Dy(iii) complex, with the formula [Dy(Hcpt)_3_]·2H_2_O (1), has been successfully prepared *via* self-assembly between Dy(iii) ions and 2-cyano-*N*′-(1-(pyridin-2-yl)amido)acetyl (Hcpt) ligand. X-ray diffraction study shows that the Dy(iii) ion is nine-coordinated by three Hcpt ligands with a tridentate chelating mode, leading to an approximately monocapped square-antiprismatic (*C*_4v_) geometry. Magnetic data analysis demonstrates that 1 performs field-induced slow magnetic relaxation with a relaxation barrier of 97.90 K, due to the quantum tunneling effect suppressed upon a static dc field of 2000 Oe. To deeply understand the magnetic behaviors, the relaxation mechanisms and magneto-structure relationship are rationally discussed using *ab initio* calculations as well.

## Abstract 2

Reaction of Dy(iii) ion with tridentate acylhydrazone ligand leads to a field-induced Dy(iii) SIM, of which the magneto-structural correlation is elucidated by the magnetic and theoretical studies.

## Graphical abstract

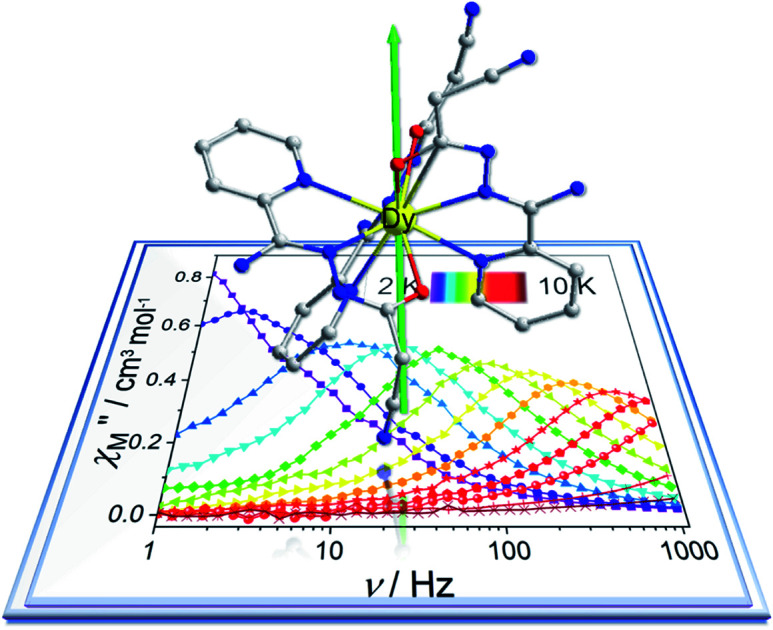

## Introduction

Since the first single-molecule magnet (SMM), Mn_12_Ac, was discovered in the 1990s, many magnetic molecules exhibiting a slow relaxation of magnetization have been synthesized and magnetically characterized, which have potentially fascinating applications in quantum computers, ultra-high-density data processing, and spintronics devices.^1–6^ In particular, the lanthanide (Ln) ions, with large ground-state spin and intrinsic magnetic anisotropy, have become excellent candidates in designing and synthesizing SMMs as magnetic centers for enhancing the magnetic inversion barrier (*U*_eff_) and blocking temperature (*T*_B_).^7–13^

The mononuclear Ln(III)-SMMs concerning mainly Dy(iii), Tb(iii) or Er(iii) ions, *etc.*,^14^ so-called single-ion magnets (SIMs), with significant single-ion anisotropy, possess unprecedented potential in boosting SIMs properties, when the coordination environments of the metal ions render strongly uniaxial magnetic anisotropies.^15^ Among the lanthanide ions, a large number of Dy-based SIMs with different symmetries have been reported, such as *D*_4d_, *D*_5h_, *D*_6h_ and *C*_∞v_,^16–19^ and displayed diverse dynamic magnetic relaxation, which attributed to the large magnetic moment with a Kramers ground-state of ^6^H_15/2_ and a large Ising-type magnetic anisotropy of Dy(iii) ion. Significantly, a dysprosium metallocene complex [(η^5^-Cp*)Dy(η5-Cp^iPr5^)][B(C_6_F_5_)_4_] (Cp^iPr5^ = penta-iso-propylcyclopentadienyl and Cp* = penta-methylcy clopentadienyl), exhibited the highest energy barrier of SIMs, has reached 1541 cm^−1^, as well as the highest magnetic blocking at a temperature up to 80 K, its further spurred activity in this area of mononuclear Ln(III) complexes.^20^ Obviously, rationalizing the coordination environment and local symmetry of Dy(iii) ions is expected to be effective for building high-performance SIMs, with significant *U*_eff_ and *T*_B_.

Existing researches proposed that high-performance lanthanide SIMs were also observed in the family with low coordination symmetries.^21^ Therefore, Ln(III)-based SIMs have attracted extensive interest as these single slow relaxation lanthanide centers are the simplest model systems allowing fundamental research on magnetic relaxation dynamics. Recent efforts have also been engaged in the complete comprehension of magneto-structural relationship and detailed theoretical elucidations of the dynamic magnetic.^22^ Combining the design, synthesis, magnetic characterization and theoretical calculation of the complex, systematic study on magnetic dynamic behavior and relaxation mechanism will play an important role in promoting the development of single-molecule magnets.^23^

**Scheme 1:**
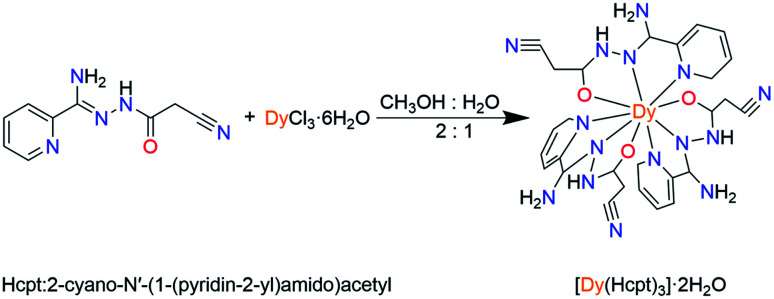
Synthesis of complex 1.

The ligand Hcpt, 2-cyano-*N*′-(1-(pyridin-2-yl)amido)acetyl, is chosen due to: (1) the ligand exhibits rigid backbone possesses and quasilinear three N, O, N donors; (2) the ligand exhibits keto–enol tautomerism and the possibility of electrostatic interactions with similar moieties, favoring the organization of the molecules on the macromolecular scale. Herein, a mononuclear complex, [Dy(Hcpt)_3_]·2H_2_O (1), was synthesized by the reaction of the tridentate Hcpt ligand with Dy(iii) ions (Scheme 1). The nine-coordinate Dy(iii) ion presents a configuration of monocapped square-antiprismatic (*C*_4v_) geometry. Accordingly, the uniaxial magnetic anisotropy, magneto-structural correlation and relaxation mechanism are investigated by magnetic experiments and *ab initio* calculations.

## Experimental

### Materials and physical measurements

All reagents and starting solvents achieved from commercial channels were of analytical grade. The C, H, and N microanalyses were implemented on a PerkinElmer 2400 CHN analyzer. Fourier transforms infrared (FT-IR) spectra were recorded in the range of 400–4000 cm^−1^ using KBr pellets on an EQUINOX55 FT/IR spectrophotometer. The phase purity of the bulk or polycrystalline samples were confirmed by powder X-ray diffraction (PXRD) measurements executed on a Rigaku RU200 diffractometer at 60 kV, 300 mA, and Cu Kα radiation (*λ* = 1.5406 Å), with a scan speed of 5° min^−1^ and a step size of 0.02° in 2*θ*. Magnetic measurements were accomplished using a Quantum Design MPMS-XL7 superconducting quantum interference device (SQUID) magnetometer on polycrystalline samples (restrained in eicosane to prevent torquing at high fields). The measured magnetic data were corrected for the diamagnetism of the constituent atoms using Pascal's tables.

### Synthesis of [Dy(Hcpt)_3_]·2H_2_O (1)

A mixed CH_3_OH/H_2_O solution (15 mL, 2 : 1) of triethylamine (0.007 mL, 0.05 mmol) and Hcpt (0.0613 g, 0.30 mmol) was stirred for 0.5 h, and then Dy(NO_3_)_3_·6H_2_O (0.0913 g, 0.20 mmol) was added. The mixture above was allowed to be stirring for 24 h at normal temperature. Primrose yellow crystals of 1 were isolated by slow evaporation of the filtrate after a week (yield 72%, based on Dy^3+^). Elemental analysis: (%) calcd for C_27_H_28_DyN_15_O_5_ (805.14): C, 58.34; N, 3.24; H, 4.43. Found: C, 58.05; N, 3.10; H, 4.25. Main IR (KBr): 3068 (w), 1601 (s), 1527 (s), 1311 (s), 1276 (s), 1242 (m), 1167 (w), 1142 (m), 1050 (s), 747 (s), 712 (s), 689 (m).

### X-ray crystallography

The X-ray experiments were implemented on a Bruker SMART APEX-CCD-based diffractometer (Mo Kα radiation, *λ* = 0.71073 Å) at low temperature. Using Olex2, the structure of 1 is solved with the ShelXT^24^ structure solution program by using Intrinsic Phasing, and refined with the ShelXL^25^ refinement package by using Least Squares Minimisation. All the non-hydrogen atoms are refined anisotropically. All the hydrogen atoms of complex 1 are located from difference maps by the program Olex2. Crystallographic data and refinement parameters are listed in Table 1, while selected interatomic distances and angles for complex 1 are given in Table S1.†

**Table 1:** Crystal data and structure refinement details for complex 1

	1
Empirical formula	C_27_H_28_DyN_15_O_5_
Formula weight	805.14
Crystal system	Monoclinic
Space group	*P*2_1_/*n*
*a* (Å)	16.8382(14)
*b* (Å)	9.3932(8)
*c* (Å)	21.5579(18)
*α* (°)	90
*β* (°)	112.5920(10)
*γ* (°)	90
*V* (Å^3^)	3148.0(5)
*Z*	4
*μ* (mm^−1^)	2.438
Unique reflections	5523
Observed reflections	15 595
*R*_int_	0.0578
Final *R* indices [*I* > 2*σ*(*I*)]	*R*_1_ = 0.0374; w*R*_2_ = 0.0797
*R* indices (all data)	*R*_1_ = 0.0633; w*R*_2_ = 0.1029

### Computational details

Complete-active-space self-consistent-field (CASSCF) calculations of complex 1 (see Fig. S4† for the complete structure of complex 1) extracted from the complex in the basis of single-crystal X-ray determined geometry have been carried out with MOLCAS 8.0 program package.^26^ For CASSCF calculation, the basis sets for all atoms are atomic natural orbitals from the MOLCAS ANO-RCC library: ANO-RCC-VTZP for Dy(iii); VTZ for close O, N; VDZ for distant atoms. The calculations employed the second order Douglas–Kroll–Hess Hamiltonian, where scalar relativistic contractions were taken into account in the basis set and the spin–orbit coupling was handled separately in the restricted active space state interaction (RASSI-SO) procedure. The active electrons in 7 active spaces include all f electrons (CAS (9 in 7)) for complex 1 in the CASSCF calculation. To exclude all the doubt we calculated all the roots in the active space. We have mixed the maximum number of spin-free state which was possible with our hardware (all from 21 sextets, 128 from 224 quadruplets and 130 from 490 doublets for Dy(iii)).

## Results and discussion

### Crystal structures

**Fig. 1:**
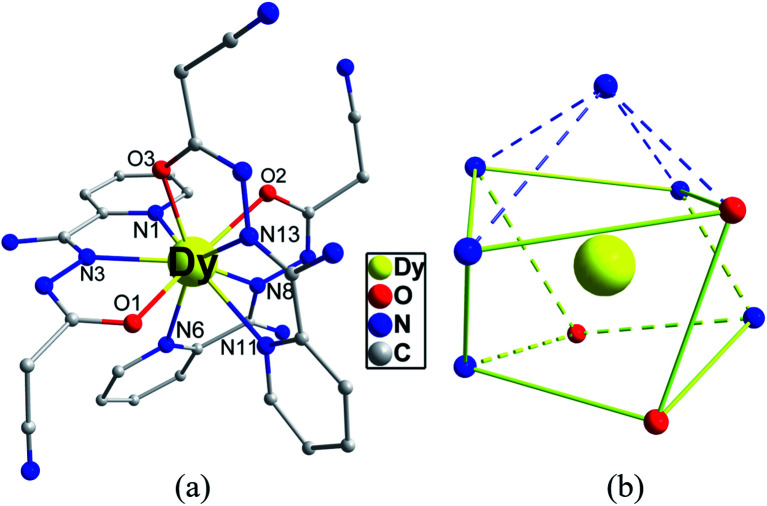
Crystal structures of complex 1 (a) and local coordination geometries of the Dy(iii) ions (b). Color code: Dy (yellow), O (red), N (blue) and C (gray). H atoms were omitted for clarity.

X-ray crystallographic analysis reveals that 1 is a mononuclear structure crystallizing in the monoclinic *P*2_1_/*n* space group. The asymmetric unit of 1 contains one independent Dy(iii) ion, three negative cpt ions and two free H_2_O molecules. The central Dy(iii) ion is nine-coordinated by three O atoms and six N atoms from three Hcpt ligands (Fig. 1a). The average Dy–O and Dy–N bond lengths are 2.378 Å and 2.538 Å, respectively. The geometrical configuration of Dy(iii) center in 1 was investigated with the SHAPE 2.1 software^27^ based on the structural parameters, the typical geometric polyhedron is depicted in Fig. 1b. In principle, the data extracted from the package tend to zero, responding to the optimal geometry, whereas a greater value presents a major deviation from the optimal polyhedron. As listed in Table S2,† the calculated values suggest that the Dy(iii) ion in 1 is best described as a monocapped square-antiprism (*C*_4v_) with moderate distortions from the ideal geometry. In addition, there are π–π interactions in 1 (Fig. S5†), yielding a three-dimensional supramolecular structure. The smallest intermolecular Dy⋯Dy separation is 9.393 Å.

### Magnetic studies

**Fig. 2:**
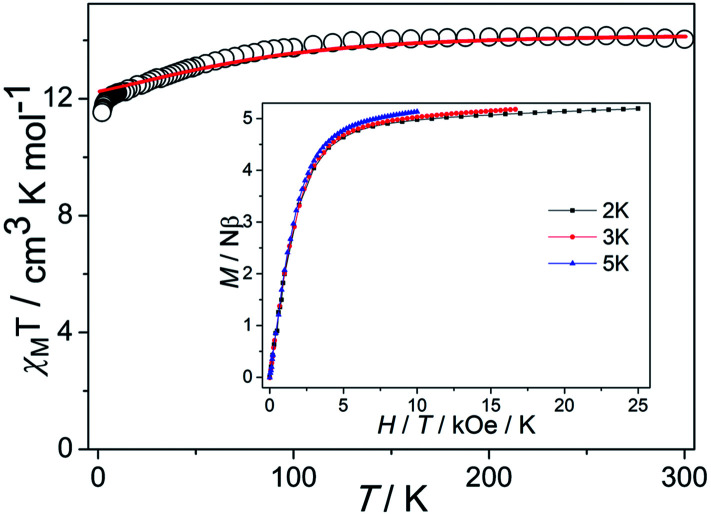
Temperature dependence of *χ*_M_*T* (solid lines represent the simulation from *ab initio* calculation). Insets: *M vs. H*/*T* plots at different temperatures.

In order to ensure the reliability of subsequent magnetic studies, the PXRD test study was carried out on the prepared polycrystalline samples (Fig. S1†). The results indicate that the powder diffraction experiment and the theoretical simulation are in good agreement, which fully confirms the purity of the sample. Direct-current (dc) magnetic susceptibilities of complex 1 were determined on polycrystalline samples in the temperature range of 2–300 K under a 1000 Oe dc field (Fig. 2). The *χ*_M_*T* value is 13.74 cm^3^ K mol^−1^ at 300 K, which is close to the expected value of 14.17 cm^3^ K mol^−1^ for one isolated Dy(iii) ion (*S* = 5/2, *L* = 5, *J* = 15/2, ^6^H_15/2_, *g* = 4/3), indicating a significant orbital contribution to the magnetic moment. For 1, the *χ*_M_*T* value stays essentially constant with only a little decrease upon cooling before steeply decreasing to 11.53 cm^3^ K mol^−1^ at 2 K. Such tendency is mainly attributed to the progressive depopulation of the Kramers doublets (KDs) of the Dy(iii) ion or weak antiferromagnetic dipolar coupling between the mononuclear groups.^28^ Although the smallest intermolecular Dy⋯Dy distance is about 9.4 Å, the neutral molecules are connected by the π–π stacking which could drive weak antiferromagnetic interactions and further have an influence on the dynamic magnetism. The *M versus H* curves were determined from 0 to 5 T at 2, 3, 5 K (Fig. S2†). At 2 K, the magnetization saturation value of the complex is 5.19 *Nβ* at 5 T, deviating significantly from the theoretical saturation value of 10 *Nβ*. In addition, the *M versus H*/*T* plots at different temperatures display non-superimposed magnetization curves, indicating the existence magnetic anisotropy and/or low-lying excited states.^29,30^

To probe the dynamic magnetic behavior for complex 1, the alternating-current (ac) magnetic susceptibilities were carried out with a 2 Oe ac field under zero field in the range of 2.0–30.0 K (Fig. S3†). Unfortunately, no out-of-phase 
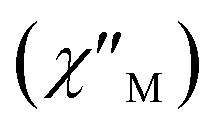
 signal was observed until the temperature drops to 2 K, illustrating 1 does not have a slow magnetic relaxation behavior under zero field, and there is a strong quantum tunneling effect of magnetization (QTM), which is probably aggravated by the intermolecular π–π stacking effects.^31^

**Fig. 3:**
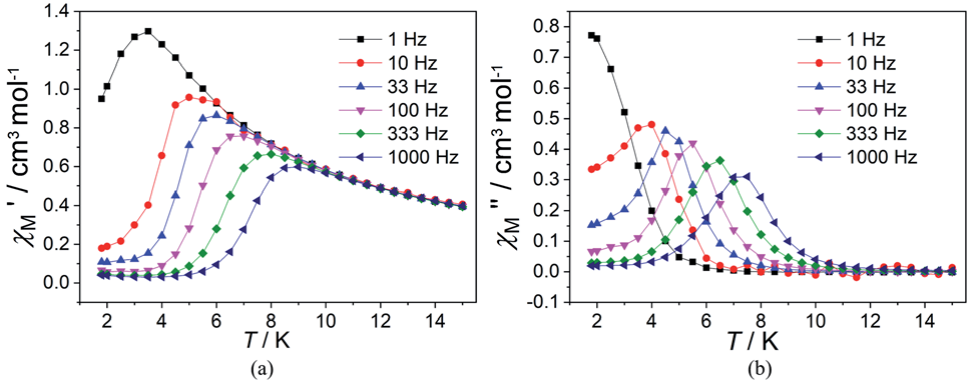
Temperature dependence of 
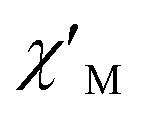
 (a) and 
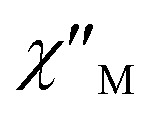
 (b) susceptibilities for 1 at applied dc fields of 2000 Oe.

**Fig. 4:**
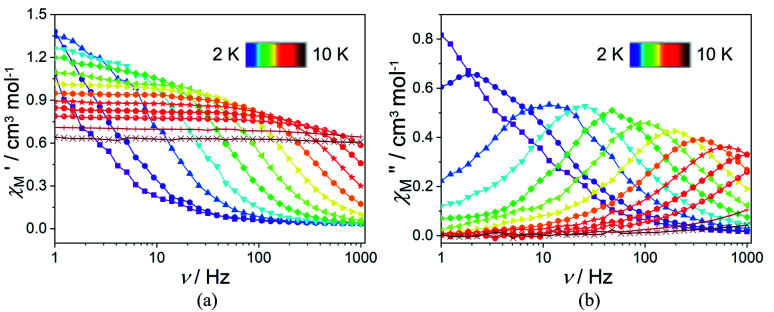
Frequency dependence of 
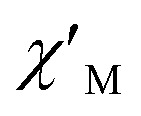
 (a) and 
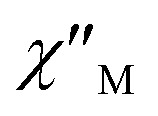
 (b) susceptibilities for 1 at applied dc fields of 2000 Oe.

**Fig. 5:**
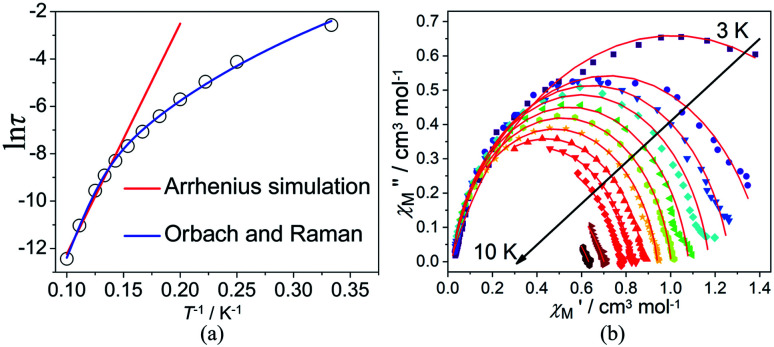
Plots of relaxation time (ln *τ*) *vs. T*^−1^ at applied dc fields of 2000 Oe (a). The red and blue lines are fitted with the Arrhenius law and multiple relaxation processes, respectively. Cole–Cole plots under 2000 Oe for 1 (b). The solid lines are the best fit to the experimental data.

In order to suppress or minimize the QTM, it is necessary to lift the degeneracy of the states to prevent the spins relaxing through tunnelling. This can be achieved by applying a dc field. Thus, the *χ*′′ susceptibilities for 1 were recorded under different magnetic fields to select a proper static field to suppress the QTM. The *χ*′′ signals with significant peak values at around 2000 Oe dc field suggest that field-induced slow magnetic relaxation and slowest relaxation operating in complex 1. The *τ vs. H* plots of 1 are correspondingly depicted (Fig. S6†). Thus, 2000 Oe was used as a suitable applied field to repress the QTM for 1. As shown in Fig. 3, the 
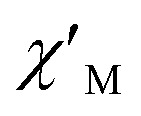
 and 
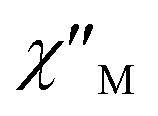
 components of complex 1 exhibit prominently temperature-dependent peaks above 1 Hz at 2000 Oe dc field, which make clearly the slow magnetic relaxation. Meanwhile, the frequency dependencies of ac data were measured from 2 to 10 K for 1 (Fig. 4). Both 
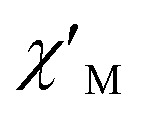
 and 
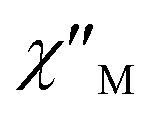
 plots of complex 1 emerge with significant frequency dependencies. As the temperature rises, the maximum points of the 
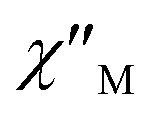
 signals in 1 smoothly move from the low frequency to the high frequency, which explicitly proclaims the probability of the relaxation through the QTM path is efficiently suppressed under the applied field. Therefore, plots of ln *τ versus T*^−1^ for 1 are expressed in Fig. 5. In the higher temperature region, the Arrhenius formula is used to linearize the data in the figure. The effective energy barrier (*U*_eff_) of 1 obtained by fitting is 94.27 K, and the value of *τ*_0_ is 2.90 × 10^−10^ s that corresponds to expected *τ*_0_ values of 10^−6^ to 10^−11^ for SMMs.^32^ It is worth noting that the data in Fig. 5a bends as the temperature decreases, due to the existence of other relaxation processes.^33^ Thereby a model including two possible relaxation processes, *i.e.*, Raman and Orbach mechanisms, is employed to analyze the contribution to the relaxation in 1 by using (1):1
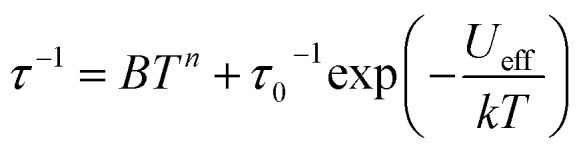


To avoid overparametrization, the *U*_eff_ and *τ* values extracted from the first fitting are properly fixed to obtain an optimal fitting curve. The fitting reproduces the experimental data very well, obtaining the parameters *B* = 0.017, *n* = 6.01, *τ*_0_ = 3.57 × 10^−10^ s, *U*_eff_ = 97.90 K. It is declared that Orbach and optical acoustic Raman-like mechanisms are synergistically responsible for the overall relaxation behaviors. ln *τ* has an excellent linear correlation in the higher temperature range, signifying that the former dominates in the high temperature regime, whereas the latter prevails in the low temperature area.^34^

Cole–Cole diagrams of 
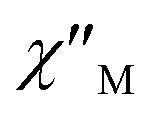
*versus*
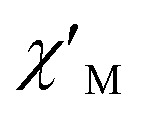
 for 1 have been obtained with the simulation of a generalized Debye function (Fig. 5b).^35^ The *α* parameters are below 0.35 at the temperature region of 3–10 K, due to the existence of Orbach and Raman processes (Table S3†).

For comparison, structural and magnetic parameters of some known nine-coordinated mononuclear Dy(iii) SMMs are summarized in Table S4.† The configurations of these nine-coordinated complexes are of *D*_3h_, *C*_4v_ or *C*_s_, exhibiting slow magnetic relaxation under a zero/non-zero dc field. The energy barrier of 97.90 K for 1 is comparatively intermediate to most nine-coordinated Dy-SMMs. The result of analyzed magnetostructural indicates that the shorter the shortest bond length and the greater the bond length difference, the better the performance of the SMMs can be obtained, suggesting the uneven bond length distribution acts the building of high-performance SIMs, as observed in the previous literature.^36^

### Theoretical investigation

**Fig. 6:**
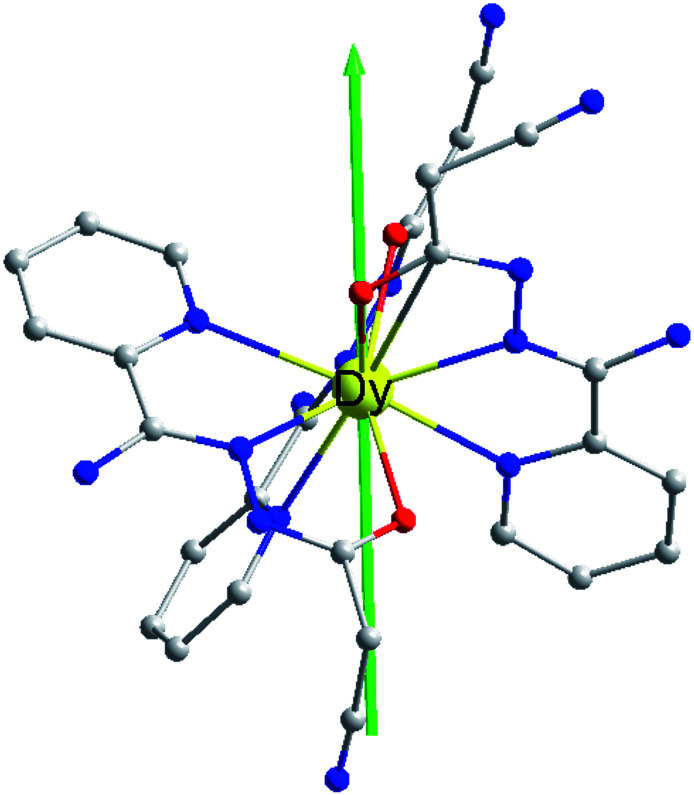
Orientation of the local main magnetic axes of the ground Kramers doublet on Dy(iii) ions.

In order to explore the slow magnetic relaxation mechanism of complex 1, *ab initio* calculations were performed. Complete-active-space self-consistent field (CASSCF) calculations on individual Dy(iii) fragments of complex 1 on the basis of X-ray determined geometries have been carried out with MOLCAS 8.0 program package.^37^ The energy levels (cm^−1^), *g* (*g*_*x*_, *g*_*y*_, *g*_*z*_) factors, and the predominant *m*_J_ values of the lowest eight Kramers doublets (KDs) of complex 1 are shown in Table 2. The ground doublet is highly axial (*g*_*x*_ = 0.015, *g*_*y*_ = 0.040, *g*_*z*_ = 19.624), which explains the prominent SMM behaviour of 1 at low temperatures. However, the *g*_*x*_ and *g*_*y*_ are not negligible for 1, which may induce the presence of the QTM effect.^38^ The calculated and experimental *χ*_M_*T* and *T* curves of 1 are shown in Fig. 2, where the fits are basically close to the experimental data. The magnetic easy axis of the ground KD in 1 lies between the Dy–O2 and Dy–O3 bonds (Fig. 6).

**Table 2:** Calculated energy levels (cm^−1^) and *g* (*g*_*x*_, *g*_*y*_, *g*_*z*_) tensors of the lowest Kramers doublets (KDs) of the Dy(iii) for complex 1

KDs	1		
	*E*/cm^−1^	*g*	*m*_J_
1	0.00	0.015	±15/2
		0.040	
		19.624	
2	140.57	0.305	±9/2
		0.852	
		17.794	
3	181.07	0.013	±13/2
		0.965	
		16.090	
4	229.00	2.039	±11/2
		3.017	
		11.073	
5	284.06	7.610	±7/2
		6.668	
		2.692	
6	313.87	0.224	±5/2
		1.926	
		14.999	
7	366.79	3.066	±3/2
		4.327	
		12.640	
8	468.37	0.286	±1/2
		0.626	
		18.506	

**Fig. 7:**
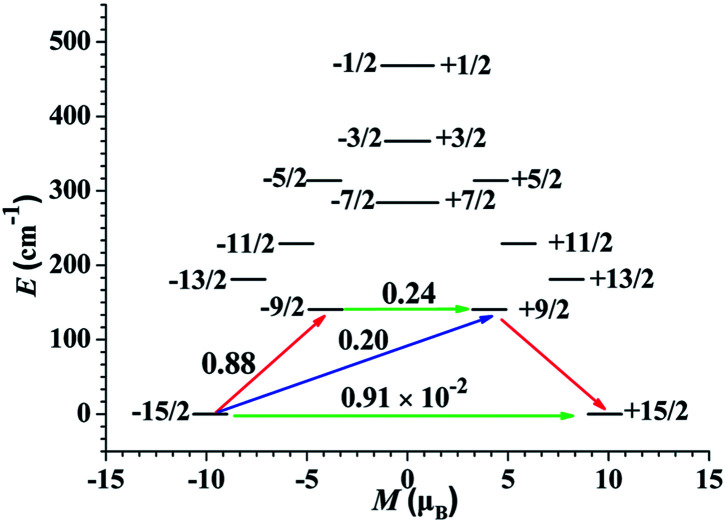
The magnetization blocking barriers for single Dy(iii) sites of 1. The thick black lines represent the KDs as a function of their magnetic moment along the magnetic axis. The green lines correspond to the diagonal matrix element of the transverse magnetic moment; the blue lines represent Orbach relaxation processes. The path shown by the red arrows represents the most probable path for magnetic relaxation in the corresponding complex. The numbers at each arrow stand for the mean absolute value of the corresponding matrix element of the transition magnetic moment.

To deeply study the principle of the relaxation process, the effective relaxation path of complex 1 from the maximum magnetization state of the doublet ground state to the time reversal state accompanied by the reverse magnetization was studied (Fig. 7). The transverse magnetic moments of the Kramer's doublets in the ground spin–orbit states arise to be modest in complex 1, which are about 10^−2^*μ*_B_, revealing that the diagonal quantum tunneling is operational for the Kramer's doublets in the ground state. The transversal magnetic moment in the first excited states of complex 1 is 0.24 *μ*_B_, and therefore, allowing fast QTM in first excited KDs. This is reflected by the corresponding wave function analysis of the KDs. In the case of 1, the ground KDs are found to consist of *m*_J_ = 15/2 (94%) and the strong mixing state is observed in the first excited KDs (Table S5†). In principle, *U*_eff_ based on the thermally activated Obach relaxation mechanism fitting of Ln(III) ions corresponds to the energy gap between the ground state and the first excited state.^39^ The energy of the first excited state is 140.57 cm^−1^ for 1, which is larger than the fitted *U*_eff_, indicating that the relaxation mechanism should not be the common Orbach process. The difference between the experimental and theoretical values may be due to the coexistence of various relaxation processes.^40^

The calculated crystal-field (CF) parameters *B*_*kq*_ for 1 are shown in Table S6,† where the absolute axial parameter *B*_20_ of 1 is slightly larger than the nonaxial CF parameters *B*_*kq*_. with *q* ≠ 0. The orientation of the principal axis is significantly determined by the bond length and the charge distribution around the first coordination sphere of dysprosium center. Therefore, the charge distribution of the central Dy(iii) ion and its surrounding atoms was calculated and analyzed (Table S7†). The low-lying ground state is more inclined to the ligand field where the negative charge is distributed in the axial direction, which can effectively reduce the repulsion between the f-electron cloud and the ligand and make the |±15/2> Kramers doublet state very much stable, and eventually produce strong magnetic anisotropy.^41^ The N atoms of complex 1 contribute the lowest average negative charge and the charge distributions on the three O atoms are evidently more negative than that of N atoms, clarifying that the oxygen-containing linkers exclude the f-electron cloud more intensively than the nitrogen-containing organics and yielding the dramatically diverse configuration of the static potential. Indicating that the |±15/2> Kramers doublet state is unstable under the ligand field, resulting in a weaker easy-axis ligand field. Combined with the analysis results of the magnetization blocking energy barrier in Fig. 7, it is proved that only when the low-lying ground state |±15/2> Kramers doublet state is completely occupied, the obvious easy axis anisotropy may occur.^42^

## Conclusions

In summary, this work reports the synthesis and characterization of a mononuclear Dy(iii) complex based on a tridentate ligand Hcpt. The crystal structure of the complex exhibits that the central Dy(iii) atom is in the nine-coordinated environment with a distorted monocapped square-antiprismatic (*C*_4v_) geometry. Magnetic characterization unveils that the performance of single-molecule magnet is absent in 1 under zero dc field. Whereas it features a significant slow magnetic relaxation process with an anisotropy barrier of 97.90 K upon a 2000 Oe dc field. The magnetic behavior of 1 has been systematically explored by *ab initio* calculations. The difference between the experimental and theoretical barriers may be due to the coexistence of various relaxation processes.

## Conflicts of interest

There are no conflicts of interest to declare.

## Supplementary Material

RA-012-D2RA02260E-s001

RA-012-D2RA02260E-s002
