# Supplementary material for: A mononuclear nine-coordinated Dy(iii) complex exhibiting field-induced single-ion magnetism behaviour
Source: RSC Adv. 2022 May 10;12(22):13992–8. doi: 10.1039/d2ra02260e (PMC9089352; doi:10.1039/d2ra02260e)
Supplement: RA-012-D2RA02260E-s001 [file RA-012-D2RA02260E-s001.pdf]

Electronic Supplementary Material (ESI) for RSC Advances.
This journal is © The Royal Society of Chemistry 2022

Supporting Information

A mononuclear nine-coordinated Dy(III) complex exhibiting field-induced single-ion magnetism behaviour

Biao Hu,[a#] Jing Xi,[a#] Peipei Cen,[b] Yan Guo,[a] Yi Ding,*[,a] Yuanyuan Qin,[a] Yi-Quan Zhang,*[,c] and Xiangyu Liu*[,a]

[a.] State Key Laboratory of High-efficiency Utilization of Coal and Green Chemical Engineering, College of Chemistry and Chemical Engineering, Ningxia University, Yinchuan 750021, China. E-mail: xiangyuliu432@126.com; yiding@nxu.edu.cn

[b.] College of Public Health and Management, Ningxia Medical University, Yinchuan 750021, China

[c.] Jiangsu Key Laboratory for NSLSCS, School of Physical Science and Technology, Nanjing Normal University, Nanjing 210023, China. E-mail: zhangyiquan@njnu.edu.cn

# These authors contributed equally to this work.

***Corresponding author**

Prof. Xiangyu Liu

E-mail: xiangyuliu432@126.com

Dr. Yi Ding

E-mail: yiding@nxu.edu.cn

Prof. Yi-Quan Zhang

zhangyiquan@njnu.edu.cn

Contents

Fig. S1 PXRD curve of complex **1**

Fig. S2 *M* vs *H* curves for **1** at basic temperatures.

Fig. S3 Temperature dependence curves of χ_M' and χ_M'' susceptibilities without dc field

Fig. S4 Calculated complete structure of complex **1**; H atoms are omitted.

Fig. S5 Packing diagram of complex **1** along b axis.

Fig. S6 Plot of τ vs. *H* for **1** under different dc fields.

Table. S1 Selected bond lengths (Å) and bond angles (°) for complex **1**.

Table. S2 Dy(III) ions geometry analysis of **1** by SHAPE 2.1 software.

Table. S3 Relaxation fitting parameters from least-squares fitting of χ(f) data under 2000 Oe dc field of **1**.

Table. S4 Structural and magnetic parameters for the reported nine-coordinated mononuclear Dy(III) SMMs.

Table. S5 In wave functions with definite projection of the total moment | *JM* > for the lowest three Kramers doublets (KDs) of the Dy(III) for complex **1**.

Table. S6 *ab initio* computed crystal-field parameters for complex **1**.

Table. S7 Natural Bond Order (NBO) charges per atoms in the ground state of complex **1** calculated within CASSCF.

References

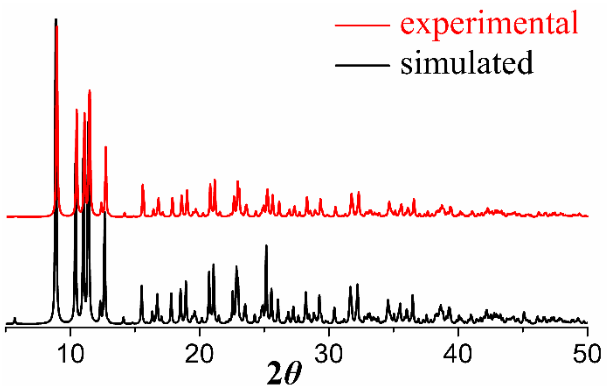

Fig. S1 PXRD curve of complex **1**

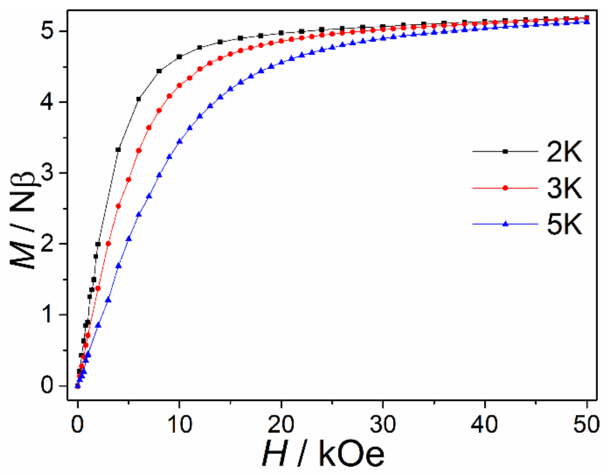

Fig. S2 *M* vs *H* curves for **1** at basic temperatures.

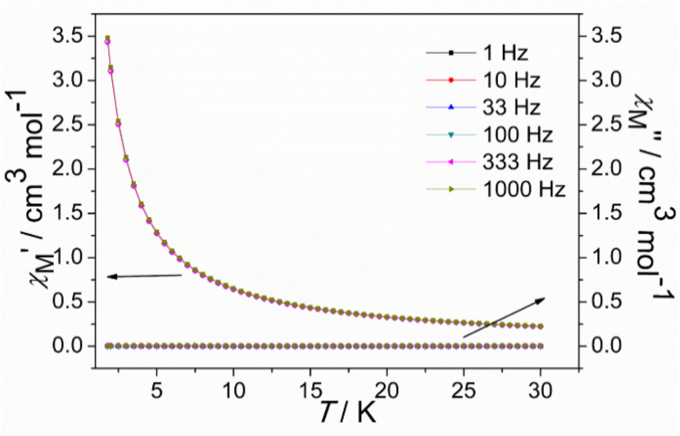

Fig. S3 Temperature dependence curves of χ_M' and χ_M'' susceptibilities without dc field

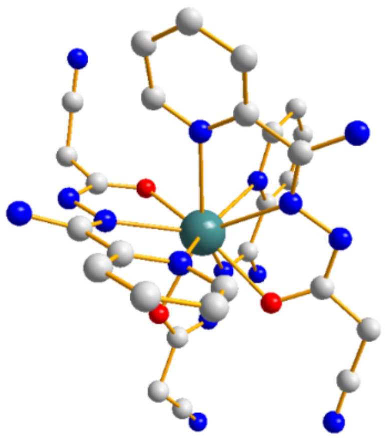

Fig. S4 Calculated complete structure of complex **1**; H atoms are omitted.

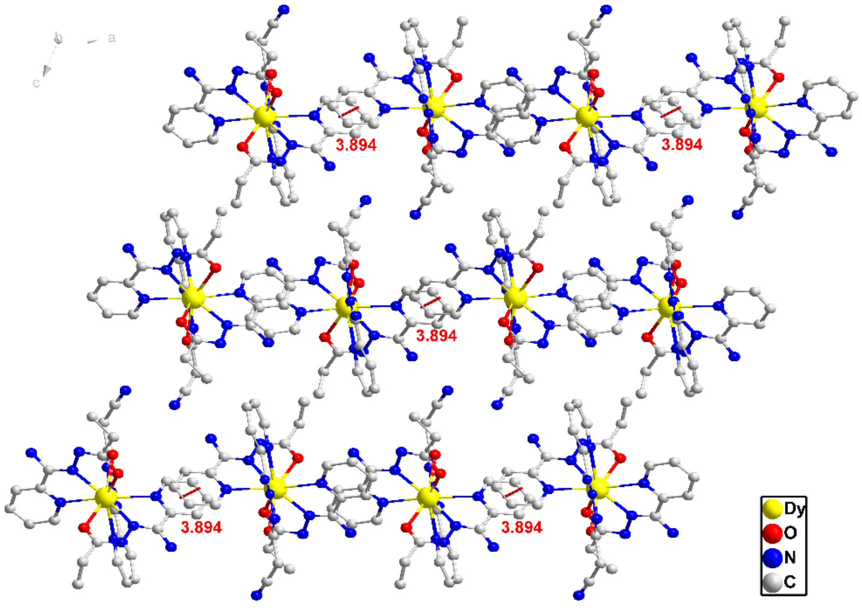

Fig. S5 Packing diagram of complex **1** along b axis.

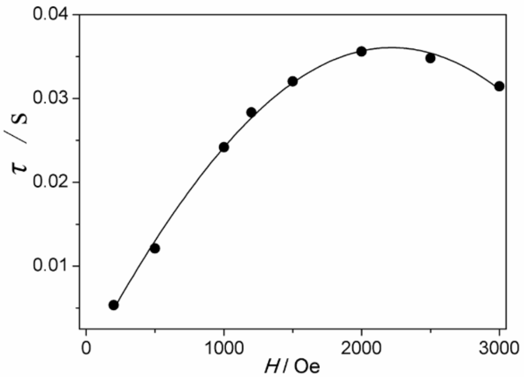

Fig. S6 Plot of τ vs. H for **1** under different dc fields.

Table. S1 Selected bond lengths (Å) and bond angles (°) for complex **1**.

Complex **1**			
Dy(1)-O(3)	2.350(4)	O(1)-Dy(1)-O(2)	150.74(15)
Dy(1)-O(2)	2.395(4)	O(1)-Dy(1)-N(3)	64.23(15)
Dy(1)-O(1)	2.390(4)	O(1)-Dy(1)-N(6)	81.14(15)
Dy(1)-N(3)	2.441(5)	O(1)-Dy(1)-N(13)	79.49(16)
Dy(1)-N(6)	2.640(5)	O(1)-Dy(1)-N(11)	72.45(15)
Dy(1)-N(13)	2.445(5)	O(1)-Dy(1)-N(1)	126.25(16)
Dy(1)-N(11)	2.619(5)	O(1)-Dy(1)-N(8)	133.29(16)
Dy(1)-N(1)	2.607(5)	N(3)-Dy(1)-N(6)	74.16(17)
Dy(1)-N(8)	2.476(5)	N(3)-Dy(1)-N(13)	123.79(18)
O(3)-Dy(1)- O(2)	74.84(15)	N(3)-Dy(1)-N(11)	132.59(16)
O(3)-Dy(1)- O(1)	91.05(16)	N(3)-Dy(1)-N(1)	62.74(17)
O(3)-Dy(1)- N(3)	74.02(16)	N(3)-Dy(1)-N(8)	124.67(17)
O(3)-Dy(1)- N(6)	147.47(16)	N(13)-Dy(1)-N(6)	142.31(16)
O(3)-Dy(1)- N(13)	64.89(15)	N(13)-Dy(1)-N(11)	62.15(17)
O(3)-Dy(1)- N(11)	126.45(15)	N(13)-Dy(1)-N(1)	139.84(17)
O(3)-Dy(1)- N(1)	82.67(16)	N(13)-Dy(1)-N(8)	111.52(17)
O(3)-Dy(1)- N(8)	135.33(16)	N(11)-Dy(1)-N(6)	81.28(16)
O(2)-Dy(1)- N(3)	132.14(16)	N(1)-Dy(1)-N(6)	76.98(16)
O(2)-Dy(1)- N(6)	124.07(16)	N(1)-Dy(1)-N(11)	147.73(17)
O(2)-Dy(1)- N(13)	71.34(16)	N(8)-Dy(1)-N(6)	62.43(17)
O(2)-Dy(1)- N(11)	95.21(16)	N(8)-Dy(1)-N(11)	73.92(16)
O(2)-Dy(1)-N(1)	78.03(16)	N(8)-Dy(1)-N(1)	74.96(16)
O(2)-Dy(1)-N(8)	63.07(16)	C(6)-N(3)- Dy(1)	126.4(4)

Table. S2 Dy(III) ions geometry analysis of **1** by SHAPE 2.1 software.

Dy(III) ion geometry analysis of **1**

```
HBPY-9       3 D7h   Heptagonal bipyramid
JTC-9        4 C3v   Johnson triangular cupola J3
JCCU-9       5 C4v   Capped cube J8
CCU-9        6 C4v   Spherical-relaxed capped cube
JCSAPR-9     7 C4v   Capped square antiprism J10
CSAPR-9      8 C4v   Spherical capped square antiprism
JTCTPR-9     9 D3h   Tricapped trigonal prism J51
TCTPR-9     10 D3h   Spherical tricapped trigonal prism
JTDIC-9     11 C3v   Tridiminished icosahedron J63
HH-9        12 C2v   Hula-hoop
MFF-9       13 Cs    Muffin

Structure [ML9 ]   HBPY-9    JTC-9    JCCU-9    CCU-9    JCSAPR-9    CSAPR-9    JTCTPR-9    TCTPR-9    JTDIC-9    HH-9    MFF-9
 ABOXIY      ,   16.576,   14.279,    8.934,    7.431,    2.529,     1.330,     3.251,     1.811,    10.676,    11.056,    1.536
```

Configuration	ABOXIY, **1**
Heptagonal bipyramid (D_{7h})	16.576
Johnson triangular cupola J3 (C_{3v})	14.279
Capped cube J8 (C_{4v})	8.934
Spherical-relaxed capped cube (C_{4v})	7.431
Capped square antiprism J10 (C_{4v})	2.529
Spherical capped square antiprism (C_{4v})	**1.330**
Tricapped trigonal prism J51 (D_{3h})	3.251
Spherical tricapped trigonal prism (D_{3h})	1.811
Tridiminished icosahedron J63 (C_{3v})	10.676
Hula-hoop (C_{2v})	11.056
Muffin (C_s)	1.536

Table. S3 Relaxation fitting parameters from least-squares fitting of χ(f) data under 2000 Oe dc field of **1**.

T(K)	χ_T	χ_S	α
2	0.035611	0.193631	0.000189
3	0.076706	0.013461	0.256001
4	0.016252	0.014854	0.165243
4.5	0.007006	0.018348	0.123448
5	0.003328	0.017357	0.109393
5.5	0.001636	0.017234	0.105143
6	0.000853	0.022761	0.098265
6.5	0.000463	0.016994	0.112061
7	0.00025	0.006492	0.123548
7.5	0.000134	1.02E-07	0.135951
8	7.05E-05	1.57E-07	0.17384
9	1.62E-05	2.32E-07	0.281026
10	4E-06	3.4E-07	0.350573

Table. S4 Structural and magnetic parameters for the reported nine-coordinated mononuclear Dy(III) SMMs.[S1]

molecular formula	$r_{shortestDy\text{-}O}$/Å	$r_{longestDy\text{-}O}$/Å	$r_{longest}$/Å	configuration	$r_{longest}$/Å-$r_{shortestDy\text{-}O}$/Å	$r_{longest}$/Å-$r_{longestDy\text{-}O}$/Å	U_{eff}/k_B/K	dc field/Oe	τ_0/s
$[Dy(H_2L)(NO_3)(H_2O)(EtOH)](NO_3)_2\cdot H_2O$[S1a]	2.322	2.533	2.533	C_s	0.211	0	20.63	1000	2.69×10^{-7}
$[Dy(Htpy)(NO_3)_2(acac)]$ [S1b]	2.257	2.529	2.533	C_s	0.276	0.004	22.7	0	6.98×10^{-7}
$[Dy(H_3daps)(H_2O)_2(NO_3)]\cdot(NO_3)\cdot$ (MeOH)[S1c]	2.305	2.53	2.532	C_s	0.227	0.002	23.8	2000	9.14×10^{-5}
$[Dy(2,3'\text{-pcad})(NO_3)\text{-}(H_2O)_4]\cdot NO_3\cdot H_2O$[S1d]	2.307	2.564	2.579	C_{4v}	0.272	0.015	24.95	1200	5.5×10^{-8}
$[Dy(L)(NO_3)(EtOH)_2]_{(SR)}$ [S1a]	2.291	2.502	2.525	C_s	0.234	0.023	28.49	1000	6.22×10^{-7}
$[Dy(H_4daps)(H_2O)_3(NO_3)]\cdot (NO_3)_2\cdot(H_2O)$ [S1c]	2.351	2.455	2.548	C_s	0.197	0.093	32.7	2000	1.82×10^{-6}
$[Dy(quinbeyz)(NO_3)_2(DMF)]$ [S1e]	2.296	2.491	2.537	C_{4v}	0.241	0.046	34.8	1000	1.4×10^{-6}
$[Dy(2,3'\text{- }Hpcad)_2(H_2O)_3]\cdot 3Cl\cdot 5H_2O$[S1d]	2.384	2.431	2.59	C_{4v}	0.206	0.159	39.2	0	3.4×10^{-6}
$[Dy(2,3'\text{-pcad})(NO_3)_2(CH_3OH)_2]$ [S1d]	2.258	2.54	2.569	D_{3h}	0.311	0.029	56.11	1200	2.9×10^{-6}
$[Dy(L)(NO_3)(EtOH)_1]_{(FR)}$ [S1a]	2.291	2.502	2.525	C_s	0.234	0.023	57.52	1000	1.53×10^{-12}
$[(C_{12}H_{10}N_5O)Dy(NO_3)_2(H_2O)_2]\cdot C_2H_5OH$[S1f]	2.319	2.538	2.607	C_{4v} 、D_{3h}	0.288	0.069	58.99	500	1.16×10^{-9}
$[Dy(HL^4)_3(NO_3)_3]$ [S1g]	2.291	2.513	2.513	C_s	0.222	0	62	2000	6.11×10^{-10}
$[Dy(HL^3)_3(NO_3)_3]\cdot CH_3CN$[S1g]	2.296	2.495	2.495	C_{4v}	0.199	0	66	2000	1.29×10^{-10}
$[Dy(HL^1)_2(NO_3)_3(CH_3OH)]$ [S1g]	2.28	2.504	2.504	D_{3h}	0.224	0	67	1000	9.72×10^{-8}
Dy $(Hcpt)_3]\cdot 2H_2O$[this work]	2.35	2.395	2.64	C_{4v}	0.29	0.245	97.90	2000	3.57×10^{-10}
$Dy(bpad)_3\cdot CH_3OH\cdot H_2O$[S1h]	2.352	2.366	2.639	C_{4v}	0.287	0.273	106.93	1200	2.28×10^{-8}
$[Dy (HL^2)_2(NO_3)_3(H_2O)]$ [S1g]	2.308	2.498	2.498	C_s	0.19	0	116	2000	8.72×10^{-11}
$[(C_{12}H_{10}N_5O)Dy(NO_3)_2(C_2H_5OH)_2]\cdot H_2O$[S1f]	2.275	2.538	2.888	C_{4v}	0.613	0.35	203.11	0	1.77×10^{-9}
$\{[Dy(CH_3OH)(NO_3)_2(tpy)]\cdot CH_3OH\}n$[S1b]	2.149	2.549	2.549	C_s	0.4	0	354.36	0	1.4×10^{-10}

H_2L=2,6-diylbis(ethan-1-yl-1- ylidene)di(isonicotinohydrazide); Hacac = acetylacetone; Htpy = 4′-(4-hydroxyphenyl)-2,2′:6′,2″-terpyridine; H_4daps = 2,6-bis(2-salicyloylhydrazonoethyl)pyridine; H_4daps = 2,6-bis(1-salicyloylhydrazonoethyl)pyridine; 2,3′-Hpcad = N^3 -(2-pyridoyl)-3- pyridinecarboxamidrazone; quinbeyz = 8-hy-droxyquinoline-2-carboxyaldehyde-(benzoyl)hydrazine; HL = N^3-(2-pyridoyl)-4- pyridinecarboxamidrazone; HL^4=1-[N-(4- iodophenyl)]aminomethylidene-2(1H)naphthalenone; HL^3=1-[N-(4- methoxy)]aminomethylidene-2(1H)naphthalenone; HL^1=1-[N-(4- cholrophenyl)]aminomethylidene-2(1H)naphthalenone; HL^2=1-[N-(4- nitrophenyl)]aminomethylidene-2(1H)naphthalenone; Hbpad = N^3 -benzoylpyridine-2-carboxamidrazone; Hcpt=2-cyano-N′-(1-(pyridin-2-yl)amido) acetyl.

Table. S5 In wave functions with definite projection of the total moment | *JM* > for the lowest three Kramers doublets (KDs) of the Dy(III) for complex **1**.

<table>
<tr><th></th><th>E/cm^{-1}</th><th>wave functions</th></tr>
<tr><td rowspan="3">1</td><td>0.0</td><td>94%|±15/2>+0.4%|±9/2></td></tr>
<tr><td>140.57</td><td>4%|±15/2>+20%|±13/2>+38%|±9/2>+30%|±7/2></td></tr>
<tr><td>181.07</td><td>2%|±15/2>+65%|±13/2>+26%|±11/2>+5%|±7/2></td></tr>
</table>

Table. S6 *ab initio* computed crystal-field parameters for complex **1**.

k	q	B_{kq}
2	-2	0.34920108741873E+00
2	-1	0.10623695699148E+01
2	0	-0.16683513847537E+01
2	1	0.22724392178449E+00
2	2	0.15351060150934E+01
4	-4	-0.10070380334743E-01
4	-3	-0.14349137952895E-01
4	-2	0.49177326240960E-02
4	-1	-0.12457495719780E-01
4	0	-0.22714814504834E-02
4	1	0.17428047599169E-01
4	2	-0.12194137289474E-02
4	3	0.24460325516696E-01
4	4	0.25313908551810E-02
6	-6	-0.13332171208156E-03
6	-5	0.79185311618547E-04
6	-4	0.13304569021568E-03
6	-3	0.46855867085394E-03
6	-2	-0.38518926294551E-04
6	-1	0.88438758948006E-04
6	0	-0.32165464267250E-04
6	1	-0.26286752285944E-03
6	2	-0.33416013164089E-04
6	3	0.54028643402664E-04
6	4	0.26863954347958E-04
6	5	0.36814372298679E-03
6	6	0.24222901262908E-03

Table. S7 Natural Bond Order (NBO) charges per atoms in the ground state of complex **1** calculated within CASSCF.

	**1**
Dy	2.4555
O1	-0.8160
O2	-0.7834
O3	-0.8132
N1	-0.3561
N2	-0.3470
N3	-0.3289
N4	-0.3351
N5	-0.3325
N6	-0.3875

References

S1. (a) J. Wang, H. Wang, Y. Ma, J. Tang, L. Li, Q. Wang, B. Zhao, P. Cheng, and J. Ma, *Cryst. Growth Des.*, 2019, **19**, 3365-3371; (b) Y. Li, Y. You, P. Zhao, *Inorg. Chem.*, 2021, **60**, 11419-11428. (c) A. K. Mondal, S. Goswami, and S. Konar, *Dalton Trans.*, 2015, **44**, 5086-5094; (d) L. Sun, S. Zhang, C. Qiao, S. Chen, B. Yin, W. Wang, Q. Wei, G. Xie, and S. Gao, *Inorg. Chem.*, 2016, **55**, 10587-10596; (e) C.-L. Ji, Y.-X. Jiang, J.-C. Zhang, Z.-Y. Qi, J.-J. Kong, and X.-C. *Inorg. Chem.*, 2018, **644**, 1635-1640; (f) L. Sun, S. Zhang, Z. Jiang, Q. Yang, S. Chen, Y. Zhang, W. Wang, Q. Wei, and G. Xie, *Dalton Trans.*, 2017, **46**, 11159-11165; (g) H. Yang, S.-S. Liu, Y.-S. Meng, Y.-Q. Zhang, L. Pu, X. Wang, and S. Lin, *Dalton Trans.*, 2022, **51**, 1415; (h) X. Liu, X. Ma, W. Yuan, P. Cen, Y.-Q. Zhang, J. Ferrando-Soria, G. Xie, S. Chen, and E. Pardo, *Inorg. Chem.*, 2018, **57**, 14843-14851;
